# The role of nurse practitioners in delivering rheumatology care and services: Results of a U.S. survey

**DOI:** 10.1002/2327-6924.12525

**Published:** 2017-10-04

**Authors:** Lydia Riley, Cindy Harris, Michele McKay, Sue Ellen Gondran, Paula DeCola, Arif Soonasra

**Affiliations:** ^1^ American Association of Nurse Practitioners Austin Texas; ^2^ Pfizer, Inc. New York New York; ^3^ Pfizer, Inc. Collegeville Pennsylvania

**Keywords:** Nurse practitioners, advanced practice nurse, role of advanced practice nurse, rheumatoid arthritis, primary care

## Abstract

**Background and purpose:**

Rheumatoid arthritis (RA) is a chronic autoimmune disease characterized by inflammation, pain, joint stiffness, and progressive joint destruction. An increased demand for rheumatology healthcare professionals is anticipated in coming years; utilizing more nurse practitioners (NPs) in rheumatology may help meet this demand, and improve early detection and diagnosis of RA.

**Methods:**

The American Association of Nurse Practitioners surveyed, via e‐mail, members who were working in primary care settings to understand their educational and professional needs to help manage their patients with RA. Respondents were surveyed about their NP certifications, patient panel, information received from rheumatologists on shared patients, RA tools or resources that would be helpful, confidence in diagnosing and managing patients with RA, interest in learning about particular topics regarding RA medications, and preferences for exchanging educational information with their professional colleagues.

**Conclusions:**

The results from this survey indicate that the role of NPs in managing RA could be optimized by improved communication with treating rheumatologists, access to educational tools and resources, and further education and training in the management of RA.

**Implications for practice:**

NPs in primary care can fill a resource gap and provide access to health care for patients with RA.

## Introduction

Rheumatoid arthritis (RA) is a chronic autoimmune disease characterized by inflammation, pain, joint stiffness, and progressive joint destruction. Over time, dysregulation of the inflammatory process because of RA results in damage to a patient's joints, causing pain and loss of physical function with subsequent impaired health‐related quality of life (Strand & Khanna, 2010). The global prevalence of RA in 2010 was estimated at 0.24%, with higher rates in North America (0.44%), and rates are expected to rise in line with an aging population (Cross et al., 2014). Although RA occurs across all ethnicities and social backgrounds, women are more than twice as likely to be affected as men (Cross et al., 2014).

Expanded treatment options for RA, including biologic disease‐modifying antirheumatic drugs (bDMARDs), and therapeutic strategies, such as “treat‐to‐target,” increase the need for patient access to healthcare professionals (Solomon et al., 2014). Over the next decade, the demand for healthcare professionals in rheumatology is expected to increase for several reasons, including higher prevalence of RA, an aging population, and treatment approaches that increase the need for time spent with a healthcare professional (Coates & Helliwell, 2016; Deal et al., 2007; Myasoedova, Crowson, Kremers, Therneau, & Gabriel, 2010). Utilizing more nurse practitioners (NPs) in rheumatology is one suggested strategy that could help healthcare systems adapt to this paradigm (Deal et al., 2007). In addition, it is now established that prompt treatment with conventional synthetic (cs)DMARDs can beneficially alter the course of RA (Kyburz, Gabay, Michel, & Finckh, 2011), but failing to recognize early or more insidious symptoms in patients who present in primary care settings can hinder swift referral and treatment (Bykerk & Emery, 2010; Raza et al., 2005; van der Linden et al., 2010). As well as aiding early RA detection, referral, and diagnosis rates, NPs in primary care are well situated to provide patient education, which has a positive effect on the health and quality of life of patients with RA (Swanson & Pfenning, 2011). Currently, there are more than 234,000 NPs licensed in the United States; 95.8% prescribe medication (NPs are licensed to prescribe medication in all states and the District of Columbia) and the majority (61.4%) see at least three patients an hour (American Association of Nurse Practitioners [AANP], 2017a). NPs are certified in a range of clinical areas; 89.2% of NPs are certified in an area of primary care, and around half (49.9%) hold hospital privileges (AANP, 2017a).

NPs have been working in the field of rheumatology for more than 30 years (Hooker, 2008). They are involved with a broad range of responsibilities, including prescribing both csDMARDs and bDMARDs (Solomon et al., 2014), and can provide particular inputs and insights into the management of patients with RA (Rauch, Kirchberger, Boldt, Cieza, & Stucki, 2009). However, rapid changes in the RA therapeutic landscape necessitate a revolution in the role and knowledge of the NP seeing patients with RA (Coughlin, 2008). A 2012 survey of NPs and physician assistants working in rheumatology in the United States reported that, of the 174 respondents, only 53% had received any formal rheumatology training, although 69.5% of NP respondents had their own panel of patients with RA (Solomon et al., 2014). It is envisaged that NPs working in primary care may also have specific professional and educational needs in order to fully optimize their role of providing evidence‐based care to patients with RA, helping to decrease disease exacerbations and improve quality of life.

## Methods

The AANP is the largest and only full‐service national professional membership organization for NPs of all specialties (AANP, 2017b). The AANP developed a survey and administered it to their members who worked in a primary care setting and managed patients with RA, with the aim of understanding their educational and professional needs in rheumatology.

### Survey design and study population

The survey tool for the RA educational needs assessment was developed and validated by AANP to meet the goal of understanding the educational and professional needs of NPs related to RA. Several of the questions were taken directly, or adapted from, prior AANP studies and survey instruments. As such, the survey instrument collected data that were comparable to other national NP datasets in order to determine representativeness and generalizability. All content was reviewed by a team of NP education specialists with 60 years’ combined experience in clinical practice.

**Figure 1:**
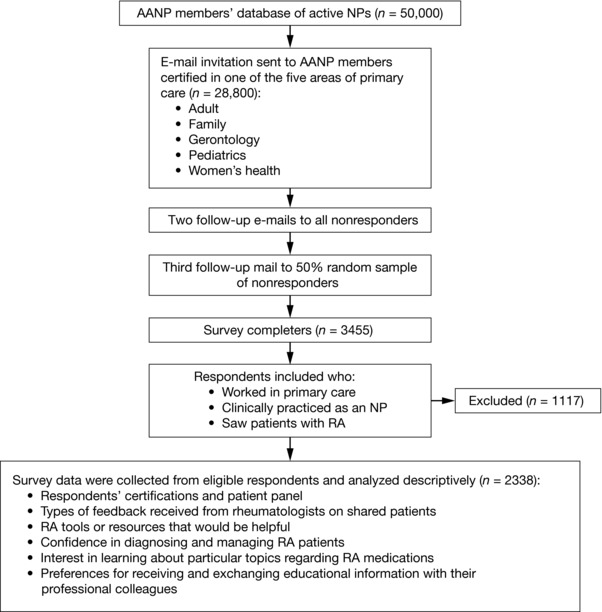
Survey study design and selection of respondents. NPs were allowed to select more than one response.

NPs who were certified in one of the five areas of primary care (adult, family, gerontology, pediatrics, and women's health) were invited to participate in the survey (Figure 1). The sample was stratified by AANP‐defined state NP practice environments to reduce bias related to scope of practice. For convenience, respondents were self‐selected, but were required to: (a) work in primary care; (b) clinically practice as an NP; and (c) see patients with RA.

Respondents were asked for information relating to their NP certifications, patient panel, types of feedback received from rheumatologists on shared patients, RA tools or resources they would find helpful, confidence in diagnosing and managing RA patients, interest in learning about particular topics regarding RA medications, and preferences for receiving and exchanging educational information with their professional colleagues. The survey consisted of six questions about the NP respondents’ clinical practice in RA, and used a combination of multiple choice questions, rating scales, and space to provide “other” responses as free text. Respondents could select more than one answer for the following questions: “Which types of feedback do you typically receive from the rheumatologist regarding your shared RA patients?” “What types of tools or resources would you find helpful in managing RA patients?” “Which of the following topics are you interested in learning more about regarding RA medications?” “Which of the following ways do you prefer to receive and exchange information with your professional colleagues?”

### Data collection and analyses

The sample for the survey was taken from the AANP's membership database, which consisted of approximately 50,000 active NP members at the time of the study. Only members who approved the use of their contact information for AANP research were included. NPs who were invited to participate in the survey were sent initial e‐mail invitations and two follow‐up e‐mails (Figure 1). Mail invitations were subsequently sent to a 50% random sample of the remaining nonresponders.

Responses were completed online or by mail and subsequently entered into an online database by data entry associates at AANP. The resulting data file was cleaned and coded in SPSS V.22, and the data analyzed using SPSS V.22 by the AANP Research Department. Responses were analyzed descriptively.

## Results

### Respondents

Between November 2015 and January 2016, 28,088 e‐mails were delivered to AANP members inviting them to participate in the survey (Figure 1). In total, 3455 (12.3%) member NPs responded to the survey; 1117 individuals who attempted to take the survey were screened out because their responses indicated that they did not meet one or more of the inclusion criteria (Figure 1); responses from 2338 (8.3%) AANP members who worked in primary care were included in the analysis.

Of the respondents who provided data on the length of time that they had been practicing NPs (*n* = 2307), the mean duration of practice was 10.5 years (standard deviation [*SD*]: 8.3 years; range: 1–46 years). Respondents (*n* = 2301) practiced in all 50 U.S. states and the District of Columbia. The majority of 2273 respondents were certified as Family (76.4%) or Adult (17.9%) NPs. The mean number of patients for which respondents (*n* = 2055) were accountable on an ongoing basis was 938 (*SD*: 1373.1; range: 0–10,000), 8.2% of whom had RA (*n* = 1951). According to 2038 respondents, the majority of their patients with RA (64.5%) were managed by a rheumatologist.

### Receiving feedback from rheumatologists

**Figure 2:**
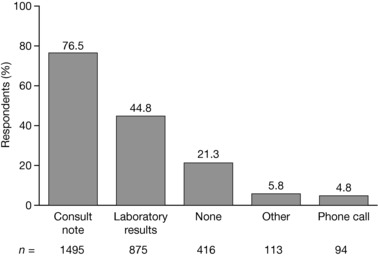
Types of feedback typically received by NPs (*N* = 1955) from rheumatologists regarding shared patients with RA. NPs were allowed to select more than one response.

Of the 1955 responses to the question, “Which types of feedback do you typically receive from the rheumatologist regarding your shared RA patients?” the majority (*n* = 1495; 76.5%) of NPs indicated that they received consult notes sent via fax, secure mail, mail, electronic medical record, or other (Figure 2). However, 416 (21.3%) respondents received no feedback from the rheumatologist on shared patients.

Among those who selected “other,” several NPs indicated that they had to request patient information from the treating rheumatologist. Others shared an electronic medical record with the rheumatologist or relied on self‐reported information from their patients.

### Tools to help manage patients with RA

**Figure 3:**
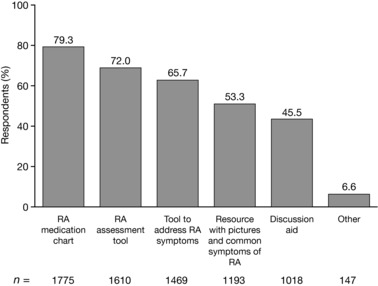
Tools and resources NPs (*N* = 2237) would find helpful in managing patients with RA. NPs were allowed to select more than one response.

Overall, 2237 NPs responded to the question, “What types of tools or resources would you find helpful in managing RA patients?” The most popular tool selected by 79.3% of respondents was an RA medication chart with indications/contraindications, adverse events, and monitoring advice (Figure 3), followed by an RA assessment tool (72.0%). Fewer than half of respondents (45.5%) expressed an interest in a decision aid to facilitate patient–provider discussion about treatment goals. Among those who selected “other,” many elaborated in the free text spaces about their interest in a tool or resource to help them assess RA patients and determine the best course of treatment.

### Level of confidence in diagnosing and managing patients with RA

**Figure 4:**
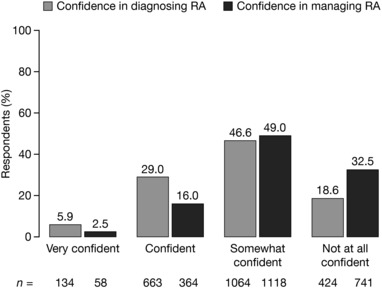
Level of confidence of NPs in diagnosing (*N* = 2285) and managing (*N* = 2281) RA.

When asked to rate their level of confidence in diagnosing RA, most respondents expressed a low‐to‐moderate level of confidence (Figure 4). Of the 2285 NPs who responded to this question, only 134 (5.9%) were very confident about diagnosing RA. “Somewhat confident” was the most popular response, selected by 1064 (46.6%) of NPs; however, 424 (18.6%) surveyed NPs indicated that they were not at all confident in diagnosing RA.

Similarly, the surveyed NPs expressed low levels of confidence in managing patients with RA (Figure 4). Of the 2281 NPs who responded to this question, only 58 (2.5%) surveyed felt very confident about managing RA in their patients. Around half (*n* = 1118; 49.0%) indicated that they were somewhat confident in managing RA; however, 741 (32.5%) were not at all confident in managing patients with RA.

### RA medication

**Figure 5:**
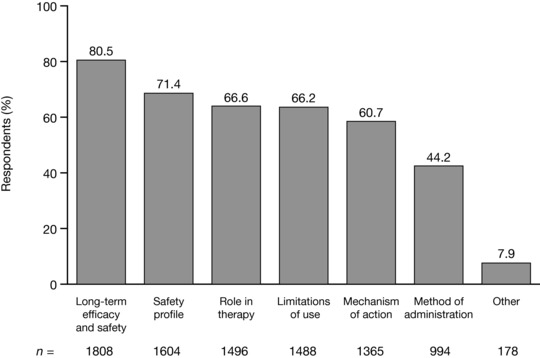
Topics regarding RA medications about which NPs (*N* = 2247) would like to learn more. NPs were allowed to select more than one response.

When the surveyed NPs were asked, “Which of the following topics are you interested in learning more about regarding RA medications?” a high level of interest was shown in a number of topics (Figure 5). The most popular topics among the 2247 responses were long‐term efficacy and safety (*n* = 1808; 80.5%) and the safety profiles (*n* = 1604; 71.4%) of RA medications. More than half of respondents also expressed interest in the “role in therapy” (66.6%), “limitations of use” (66.2%), and “mechanisms of action” (60.7%). Less than half (44.2%) expressed interest in learning more about methods of administration. Additional topics were suggested by 178 NPs who selected “other.” These frequently involved the role of RA medication in special populations (e.g., the elderly, preoperative patients, postoperative patients, pregnant women, and patients with diabetes). Other comments related to learning more about the costs associated with medications, and learning more about alternative or nonpharmacologic interventions. Eighteen open‐ended responses were recoded under “Safety profile.”

### Exchange of educational information with other healthcare professionals

**Figure 6:**
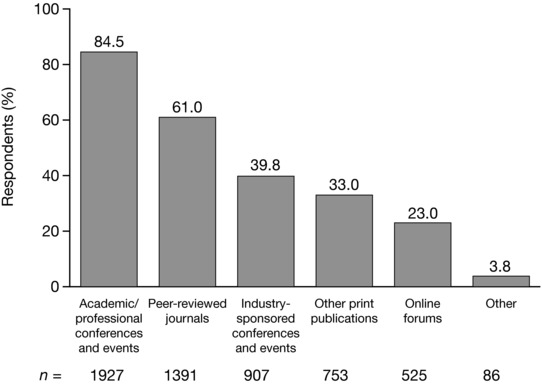
Preference of NPs (*N* = 2281) for receiving and sharing educational information with colleagues. NPs were allowed to select more than one response.

When asked to identify the ways in which they preferred to receive and exchange educational information with other healthcare professionals, of the 2281 respondents, the majority (*n* = 1927; 84.5%) selected academic conferences and events as their preferred option (Figure 6). More than half of respondents (61.0%) indicated that peer‐reviewed journals were their preferred channel. Around one in four respondents (23.0%) indicated that they preferred to receive and exchange information through online forums. E‐mail communications and online education tools were among the other channels for exchanging information suggested by respondents.

## Discussion

The roles of NPs in managing patients with RA are expected to expand and increase in the future. With this in mind, this survey of 2338 members of the AANP was conducted to help inform ways to address the professional and educational needs of NPs who manage patients with RA in a primary care setting. The quantity and quality of patient information shared with NPs by treating rheumatologists were variable. The majority of NPs did receive consult notes; however, more than 20% of respondents did not receive any patient information from rheumatologists. The role of nurses trained in rheumatology is well recognized and, although rheumatologists primarily manage RA, they do so as part of a multidisciplinary approach (Smolen et al., 2014). Multidisciplinary care is of particular importance for patients with RA and comorbid conditions, or those who have complications associated with therapy, such as infection. NPs working in primary care may well be first in line to see such patients; therefore, to facilitate a true multidisciplinary approach and optimize patient outcomes, the level of communication and information sharing between rheumatology specialists and NPs must be upheld.

In other therapy areas, algorithms are available for healthcare professionals to make decisions about when to refer patients to specialist clinicians (Tytgat et al., 2008; World Gastroenterology Organisation, 2017). Shared decision making between healthcare professionals and patients is also acknowledged as best practice in the treatment of RA (Singh et al., 2016; Smolen et al., 2017); however, patients have reported less than adequate communication with their physician (Barton et al., 2014). In this regard, NPs working in primary care are well positioned to address patient educational needs and support shared decision making (Grønning, Midttun, & Steinsbekk, 2016; Palmer & El Miedany, 2016). Shared‐decision models in RA have been developed by organizations such as the Mayo Clinic (2017), among others, and could be utilized or adapted for use by NPs. Judging by the level of responses given regarding the availability/development of tools or resources to assist with management of RA patients, the primary care NPs surveyed had a clear interest in such tools. The prospect of an RA medication chart with indications/contraindications, adverse events, and monitoring advice was particularly popular, as was an RA assessment tool. The AANP currently develops “pocket” guides for NPs to use for point‐of‐care resources and produces patient engagement flipcharts in other disease settings (AANP, 2017c); using these types of resources could satisfy the need expressed by NPs for RA management tools. The sheets of the flipchart are dual‐sided, with one side graphically illustrating the key learnings for the patient, and the other side detailing the evidence behind the points for the NP to discuss with the patient. This type of tool would serve as an engaging visual patient teaching aid, while prompting the NP to utilize the most current evidenced‐based strategies for patient‐centered RA management. Three‐sided, disease‐specific “tents” also developed by the AANP can be used passively in clinic waiting rooms to prompt patients to ask their NP or clinician about symptoms (AANP, 2017c). The ultimate goal would be to assist patients with RA to maximize their autonomy, avoid and control early disease exacerbations, and enhance their quality of life.

Primary care has been highlighted as a possible setting for delays in recognizing and referring patients (Bykerk & Emery, 2010). NPs working in primary care who responded to this survey reported low levels of confidence in their RA clinical practice capabilities, with less than half reporting that they were confident in diagnosing or managing RA. Training and education in RA could help improve awareness of the signs and symptoms of RA among primary care NPs, which may lead to earlier referrals to a rheumatology clinic. Similarly, improved knowledge of RA medication could help NPs in primary care with patients who may experience complications of treatment. Indeed, the NPs surveyed exhibited a high motivation to learn more about RA medication, with high proportions (44.2%–80.5%) expressing an interest in the topics suggested. Based on respondent preferences, professional educational exchange in RA should primarily be conducted through academic or professional conferences and events, or published in peer‐reviewed journals. A recent AANP survey showed that over a quarter (27%) of members had attended an AANP National Conference, around 5% had attended an AANP Specialty & Leadership Conference, and around 3% had attended an AANP Health Policy Conference (AANP, 2015). AANP members were also likely to have attended other specialty conferences, and local NP meetings/conferences within the last year. Access to and attendance at these events is important for all NPs. Further training in rheumatology is available to NPs via the American College of Rheumatology, which offers an online Advanced Rheumatology Course comprising a three‐track curriculum (adult, pediatric, and combined) designed to provide in‐depth information and advanced skills (American College of Rheumatology, 2017).

Results from our survey are limited by several factors. Respondents were self‐selected, and may therefore reflect an atypical sample with a greater desire and need for education and professional support than the overall NP population. As with all surveys, there is a limit to the controls available for data collection, and results may be biased by respondents’ subjectivity. Our survey focused on NPs working in primary care who saw patients with RA in that setting. Results may be different if NPs working exclusively in a rheumatology setting were surveyed. In a survey of U.K. NPs in rheumatology, active participation in the delivery of medical education, attendance at postgraduate courses, and training in practical procedures were highlighted as key factors to improve their practice (Goh, Samanta, & Samanta, 2006). There are limited data from similar surveys of primary care NPs on their experiences with patients with RA. A survey of nine nurses undertaking graduate studies, who were asked to write an essay on the work‐related skills that the rheumatology nurse needs to master, identified key areas to empower nurses (knowledge about rheumatic diseases; treatments and follow‐up monitoring care; knowledge about patient education and counseling; collaboration and the ability to co‐operate; mastery of manual skills and development of quality nursing care for patients with rheumatic disease; Juhola, Kukkurainen, & Suominen, 2007); however, the surveyed population of nine nurses may not be comparable to the audience of NPs with varying levels of clinical expertise in this study. Furthermore, although the free text “other” responses given by the respondents in response to the survey questions were insightful, they were difficult to quantify because of the subjective nature of the responses.

Given the responses received, further investigation is warranted into how NPs in primary care perceive their role in rheumatology changing and details of specific training requirements in RA also need to be defined. In particular, it would be interesting to determine whether NPs would take advantage of any specialized rheumatology training or mentoring (e.g., 1‐ to 2‐year NP Fellowships in RA), and whether it would increase their confidence in managing and monitoring patients on RA medications, including csDMARDs, targeted synthetic DMARDs, and bDMARDs.

Healthcare systems are faced with increasing demands. Changing demographics, with an aging population and an increasing burden of chronic diseases, including RA, along with the evolution of proposed changes in the Affordable Care Act, which aims to provide improved access to health care, will consequently increase patient demand for services (Iglehart, 2013). Expanding the role of NPs in RA management provides a solution to this potential crisis (Solomon et al., 2014). The value of NPs was demonstrated in an observational cohort in seven U.S. rheumatology centers, where patients seen in practices with NPs or physician assistants had disease activity control that was not inferior to, and may have been slightly better than, practices using rheumatologists only (Solomon et al., 2015). It is important to note, however, that because of the complexities in diagnosing and treating RA and other rheumatic diseases, all primary care providers, including NPs, must be trained in recognizing the symptoms of these diseases, and must know when to promptly refer to a rheumatology specialist. Primary care providers, even when not directly involved in rheumatology, must also understand the effects of treatments, including csDMARDs, bDMARDs, targeted synthetic DMARDs, and other immunomodulators, on body systems that are treated in primary care, and would benefit from having resources regarding the different treatments available for reference.

In conclusion, in a primary care setting, NPs can allow first‐line access to a healthcare professional for patients with RA, providing clinical expertise, diagnostic skills, therapeutic interventions, education, and counseling. The results from this survey indicate that their contribution could be further enhanced by improved communication with treating rheumatology specialists and access to educational tools and resources. Further education in RA and RA treatment, and the development of rheumatology specialty curricula, could support NPs in optimizing the management of RA in the future.
